# Environment-Friendly Biodiesel/Diesel Blends for Improving the Exhaust Emission and Engine Performance to Reduce the Pollutants Emitted from Transportation Fleets

**DOI:** 10.3390/ijerph17113896

**Published:** 2020-05-31

**Authors:** Amit Kumar Sharma, Pankaj Kumar Sharma, Venkateswarlu Chintala, Narayan Khatri, Alok Patel

**Affiliations:** 1 Department of Chemistry and Biofuels Research Laboratory, Centre for Alternate Energy Research, R&D, University of Petroleum and Energy Studies, Dehradun 248007, Uttarakhand, India; 2 Department of Mechanical Engineering and Biofuels Research Laboratory, Centre for Alternate Energy Research, R&D, University of Petroleum and Energy Studies, Dehradun 248007, Uttarakhand, India; narayan@ddn.upes.ac.in; 3 School of Engineering and applied Sciences, National Rail and Transportation institute, Deemed to be University, Vadodara 390 004, Gujarat, India; vchintala@ddn.upes.ac.in; 4 Biochemical Process Engineering, Division of Chemical Engineering, Department of Civil, Environmental and Natural Resources Engineering, Lulea University of Technology, 971 87 Lulea, Sweden; alok.kumar.patel@ltu.se

**Keywords:** microalgae, *chlorella vulgaris*, *jatropha*, *calophyllum inophyllum*, biodiesel blends, diesel engine, thermal efficiency, and emissions

## Abstract

Biodiesel derived from biomass is a renewable source of fuel, and global application of biodiesel in the transport sector has rapidly expanded over the last decade. However, effort has been made to overcome its main shortcoming, i.e., efficiency and exhaust emission characteristics (NOx emissions) in unmodified diesel engines. Biodiesel combustion generally results in lower unburned hydrocarbons (HC), carbon monoxide (CO), and particulate matter (PM) in exhaust emissions compared to fossil diesel. In this study, various biodiesel blends (*Chlorella vulgaris,* *Jatropha curcus,* and *Calophyllum inophyllum*) were investigated for fuel characteristics, and engine performance with exhaust emission compared to diesel. *Chlorella vulgaris*, *Jatropha curcus,* and *Calophyllum inophyllum* biodiesel were synthesized by the acid–base transesterification approach in a microwave reactor and blended with conventional diesel fuel by volume. The fuel blends were denoted as MB10 (90% diesel + 10% microalgae biodiesel), MB20 (80% diesel + 20% microalgae biodiesel), JB10 (90% diesel + 10% jatropha biodiesel), JB20 (80% diesel + 20% jatropha biodiesel), PB10 (90% diesel + 10% polanga biodiesel) and PB20 (80% diesel + 20% polanga biodiesel). Experiments were performed using these fuel blends with a single-cylinder four-stroke diesel engine at different loads. It was shown in the results that, at rated load, thermal efficiency of the engine decreased from 34.6% with diesel to 34.1%, 33.7%, 34.1%, 34.0%, 33.9%, and 33.5% with MB10, MB20, JB10, JB20, PB10, and PB20 fuels, respectively. Unburned hydrocarbon, carbon monoxide and smoke emissions improved with third-generation fuels (MB10, MB20) in comparison to base diesel fuel and second-generation fuels (JB10, JB20, PB10 and PB20). Oxides of nitrogen emissions were slightly increased with both the third- and second-generation fuels as compared to the base diesel. The combustion behavior of microalgae biodiesel was also very close to diesel fuels. In the context of comparable engine performance, emissions, and combustion characteristics, along with biofuel production yield (per year per acre), microalgae biodiesel could have a great potential as a next-generation sustainable fuel in compression engine (CI) engines compared to jatropha and polanga biodiesel fuels.

## 1. Introduction

Increasing demand of energy with limited fossil-fuel-based resources has caused a move towards sustainable and renewable sources of energy [1]. The most suitable alternative to fossil fuel is biomass-based renewable energy sources, which accounted for almost 59% of total renewable-based energy sources in 2015 for European Union [2]. Global exposure to renewable energy sources raised their consumption level in 2012 to about 19 per cent of global energy consumption. The European Union ‘s commitment to renewable energy is expected to rise by 55–75% by 2050 [2,3]. The action plan to shift towards a sustainable low-carbon economy by 2050 established key elements for EU climate action which will help the EU to become a competitive low-carbon economy by 2050 [3].

Presently the Indian government has taken unprecedented initiatives in field of biofuels to meet the huge energy demand in transportation, power generation, and agriculture. The Indian government has undertaken several policy interventions and biodiesel blending targets. For example, the biodiesel blending program (BBP) that started on 10 August 2015 in five cities has been extended to six states in the country [4]. Further, the government of India is exploring supplying a 5% biodiesel blend to bulk consumers such as Indian Railways and the defense sector [5]. However, large-scale production of biodiesel has still not started in India. On the other hand, there has been much criticism of the sustainability of first-generation biofuels crops (food versus fuel debate), net greenhouse gases (GHG) balance, and net energy balance. These criticisms have led to growing interest in second-generation and third-generation biofuels. Microalgae could be a promising option among third-generation biofuels due to its high potential of energy production per acre compared to conventional oilseed crops. Microalgae can mature within 2–3 days as compared to Jatropha, Karanja etc. (second-generation biofuels), which take 2–3 years. Microalgae have good potential for production of different types of biofuels, such as biodiesel, bioethanol, biogas, and biohydrogen. In addition, microalgae biofuels do not have any competition with agricultural land as they are aquatic species and can be cultivated in brackish or waste water. Some microalgae can double their biomass within 6–24 h and accomplish up to 50% lipid content in dry biomass [6,7]. Microalgae species are able to produce 7–30 times more oil than high-yielding terrestrial oilseed crops [8,9]. In addition to this, terrestrial crops are seasonal while microalgae can be cultivated in photo-bioreactors or open ponds throughout the year. Furthermore, microalgae require about 49–132 times less space compared to agricultural crops, such as rapeseed and soybean, for the same quantity of biodiesel production [10,11,12]. 

The performance of engines commonly depends on fuel quality. Borecki et al. proposed a capillary sensor with disposable optrode of diesel fuel quality, which shows an ability to classify known and unknown fuel samples [13]. The physicochemical properties of the biodiesel fuels generally depend upon their fatty acid methyl esters (FAMEs) compositions [14,15,16]. It has been reported that microalgae biodiesel fuels have similar favorable physiochemical properties as that of second-generation biodiesel fuels for diesel engine applications [17,18,19]. In this direction, Tüccar et al. examined the effect of algae biodiesel blends on the diesel engine performance [20] and observed a significant deterioration in brake thermal efficiency when the engine run with algae biodiesel blends. In another experimental investigation, they run a compression engine (CI) engine fueled with 10% and 20% diesel–butanol–microalgae biodiesel blends at different loads [21]. As a result, they found a slight increase in engine efficiency along with the benefit of emission reduction. Makareviciene et al. performed experiments on a multicylinder diesel engine generator using microalgae biodiesel (MB) fuel blends (70% diesel + 30% MB) and also observed a slight decrease in the engine efficiency [22]. Al-lwayzy et al. used *Chlorella Protothecoides* biodiesel blends (CP-B20, CP-B50 and CP-B100) to run a direct injection DI diesel engine [14]. Islam et al. conducted experiments on a four-cylinder turbocharged common rail DI diesel engine using algae biodiesel (10%, 20% and 50%) [23]. They suggested that 20% algae biodiesel has the closest alignment to diesel in terms of performance [23]. They assessed some of the combustion characteristics, such as in-cylinder pressure variation, indicated mean effective pressure, and rate of pressure rise for engine. However, the information on comparison of performance, combustion, and emission characteristics of microalgae biodiesel fuels in CI engines is scant in the literature. Nevertheless, there is also discrepancy on emission results from one study to another. For example, Tüccar et al. [21], Velappan et al. [24], Jayaprabakar et al. [25], Islam et al. [23], reported oxides of nitrogen (NO_x_) emission increase with the use of microalgae biodiesel in CI engines. In contrast to this, some other studies reported NO_x_ emission reduction with microalgae biodiesel blends [26,27,28]. Hence, in order to address the above research gaps, an attempt has been made to focus on comparative assessment of a single-cylinder diesel engine (3.7 kW rated power at 1500 rpm) with diesel, microalgae biodiesel (*Chlorella vulgaris* based biodiesel) blends, and jatropha and polanga biodiesel blends. The main objectives of this study are to:

- To produce microalgae, jatropha, and polanga biodiesel using acid–base transesterification;
- To assess and compare engine characteristics (performance, combustion, and emission characteristics) of microalgae (MB10 and MB20), jatropha (JB10 and JB 20) and polanga (PB10 and PB20) with base fuel, i.e., commercial diesel fuel;
- To compare third-generation biofuels (MB10, MB20) with second-generation biofuels (JB10, JB20, PB10 and PB20).

## 2. Materials and Methods

### 2.1. Production of Microalgae, Jatropha and Polanga Biodiesel

(a) Microalgae biodiesel production: A pure culture of *Chlorella vulgaris* microalgae was cultivated using commercial fertilizer as a nutrient in an open raceway pond of 1200 L working volume for 52 days at semicontinuous mode, which achieved the optimum productivity of 19.61 g/m^2^/day [29]. Microalgae biomass was harvested using alum as flocculants. After that, the biomass slurry was filtered through cotton cloth, followed by drying under sunlight and microwave treatment. About 23.15% lipid yield was extracted using chloroform:methanol (2:1 ratio) as solvent in the Soxhlet extraction method. The acid value of extracted lipid was 12.39%. Therefore, acid–base catalyzed transesterification process was employed to produce biodiesel. To reduce free fatty acids (FFAs) less than 2%, firstly acid esterification was performed using 20% methanol (*v/v*) and 1.5% H_2_SO_4_ (sulfuric acid: *v/v* ratio) at 64 °C temperature for 15 min. Now the impurities were removed from refined lipid oil by a simple gravity method. This refined lipid oil was transesterified under the following reaction conditions: catalyst 1% KOH (potassium hydroxide) and molar ratio of 1:10 of lipid to alcohol at 64 °C for 10 min. At the end of reaction, the mixture of products (biodiesel, glycerol, and excess alcohol) was allowed to separate in a separating funnel. After that, biodiesel was separated from glycerol and excess alcohol carefully and washed with (slightly acidic) hot distilled water to remove other impurities. Biodiesel is insoluble in water and easily separated from water. However, due to its hygroscopic nature, it acquires some moisture. To remove this moisture, biodiesel was treated with sodium sulphate and filtered through Whatman filter paper. The moisture-free biodiesel was stored in airtight borosilicates bottles [29].

(b) Jatropha and polanga biodiesel production methodology: jatropha and polanga biodiesel oil was procured from the local vendor in Dehradun Uttarakhand and they had free fatty acid levels of (FFA) 13.6% and 4.3% respectively. Therefore, both the jatropha and polanga biodiesel was again prepared by an acid–base catalyzed transesterification reaction process. To reduce the free fatty acids concentrations of both oils to less than 2%, the oil was esterified using methanol as cosolvent in presence of an acid catalyst (sulphuric acid). The esterified oil was allowed to separate into two layers i.e., refined oil and excess methanol with impurities. Thereafter, it was transesterified using 20% methanol (*v/v*) and KOH as catalyst at 64 °C in a microwave reactor (Table 1). The transesterified product was transferred in a separating funnel and permitted to separate into three different layers, i.e., excess methanol (uppermost layer), biodiesel (middle layer), and glycerol (lowermost layer). After separating the biodiesel layer and washing three to four times by distilled water, the moisture was again removed using sodium sulphate and a centrifuge. This moisture-free biodiesel was used to prepare fuel blends with Euro IV diesel to examine the performance and emissions. The optimized parameter used to produce the biodiesel from microalgae, jatropha, and polanga oil is shown in Table 1.

### 2.2. Physico-Chemical Properties of Fuel Blends

The biodiesels derived from jatropha, polanga, and microalgae were blended with conventional diesel in different proportions such as MB10 (10% microalgae biodiesel + 90% diesel), MB20 (20% microalgae biodiesel + 80% diesel), JB10 (10% jatropha biodiesel + 90% diesel), JB20 (20% jatropha biodiesel + 80% diesel), PB10 (10% polanga biodiesel + 90% diesel), PB20 (80% polanga biodiesel + 10% diesel). The fuel properties of pure diesel and biodiesel fuel blends (MB10, MB20, JB10, JB20, PB10, and PB20) such as density, kinematic viscosity, cloud point, flash point, oxidation stability and calorific value were analyzed as per the American Society for Testing and Materials (ASTM) standards and shown in Table 2. However, several properties, e.g., oxidation stability of biodiesel fuel blends, did not meet ASTM standards which can be improved using suitable antioxidants. Biodiesel composition was analyzed using a Nucon 5700 series Gas chromatograph equipped with flame ionization detector (FID). Nitrogen was used as carrier gas. A capillary EOX column (serial no 5061; 30 m length, 0.25 mm ID and 0.25 mm outer diameter) were used to separate and identify fatty acids [12,30,31]. *Chlorella vulgaris* biodiesel composed of 28.49% saturated fatty acid, 35.44% monounsaturated fatty acid, and 35.97% polyunsaturated fatty acid, while jatropha biodiesel was with 27.73% saturated fatty acids, 42.03% monounsaturated, and 33.48% polyunsaturated fatty acids (Table 3). In contrast, polanga biodiesel had 26.90% saturated fatty acids, 33.48% monounsaturated fatty acids, and 37.24% polyunsaturated fatty acids.

### 2.3. Engine Experimental Test Setup and Procedure

**Figure 1:**
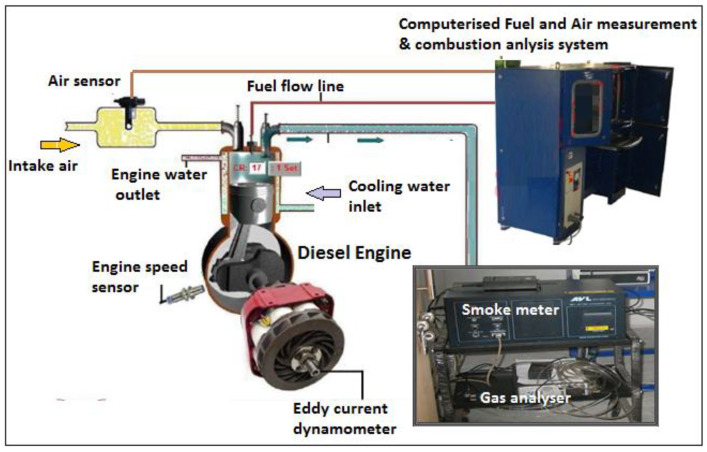
Pictorial view of experimental setup with gas analyzers and smoke meter.

The experiments were performed on a single-cylinder, four-stroke, direct injection compression ignition (CI) engine (3.7 kW) equipped with eddy current dynamometer. Technical specifications and the experimental setup of engine are shown in Figure 1 and Table 4. Emission parameters, such as hydrocarbon (HC), carbon monoxide (CO) and nitrogen oxides (NO_x_), were analyzed using a gas analyzer (Model-AVL Digas 444). A smoke meter (AVL model 437C) was used for smoke measurement. A computerized system for fuel and air consumption measurements was integrated with the test engine (Figure 1). A pressure transducer was mounted on the engine’s cylinder head for measurement of in-cylinder pressure. The engine combustion diagnosis system was used to trace the in-cylinder pressure signal with respect to engine crank angle revolutions.

To perform the experiments, the engine was run with pure diesel (D100), microalgae biodiesel blends (MB10 and MB20), jatropha biodiesel blends (JB 10 and JB 20), and polanga biodiesel blends (PB 10 and PB20) at different load conditions (20%, 40%, 60%, 80%, and 100%). The experimental test matrix is illustrated in Table 5. Emissions were represented here on mass basis and converted using Equation (1). Measurement range and resolution of various parameters are listed in Table 6. The uncertainty of experimentally measured emission parameters such as HC, CO, NOx, and smoke were determined using standard deviation method. Similarly, the uncertainty of calculated parameters such as thermal efficiency were determined using Equation (2) [32].

(1) $$\mathrm{Mass}\;\mathrm{emission}\;\mathrm{of}\;\mathrm{species}=F_{\mathrm{species}}\times \frac{{\mathrm{MW}}_{\mathrm{species}}}{{\mathrm{MW}}_{\mathrm{exhaust}\;\mathrm{gas}}}\times \frac{{\dot{m}}_{\mathrm{exhaust}\;\mathrm{gas}}}{\mathrm{Brake}\;\mathrm{power}}\times 360$$

where:

$$\begin{array}{c} F_{\mathrm{species}}=\mathrm{Mole}\;\mathrm{fraction}\;\mathrm{of}\;\mathrm{exhaust}\;\mathrm{gas}\;\mathrm{species}\;(\mathrm{CO},\;\mathrm{HC}\;\mathrm{and}\;{\mathrm{NO}}_{x})\\ \mathrm{MW}=\mathrm{Molecular}\;\mathrm{weight};\;\dot{m}=\;\mathrm{Mass}\;\mathrm{flow}\;\mathrm{rate},\;\mathrm{kg}/s \end{array}$$

(2) $$q={\left[{\left(\frac{\partial q}{\partial x_{1}}\Delta x_{1}\right)}^{2}+{\left(\frac{\partial q}{\partial x_{2}}\Delta x_{2}\right)}^{2}+\ldots \ldots \ldots +{\left(\frac{\partial q}{\partial x_{n}}\Delta x_{n}\right)}^{2}\right]}^{\frac{1}{2}}$$

### 2.4. Heat Release Rate Determination

Heat release rate (HRR) with respect to degree of crank angle (CA) during combustion of fuel (diesel/MB10/MB20) is determined using conservation of energy equation as given below (Equations (3)–(12)):

*dQ* = *dU* + *dW* + *dQ_loss_* (3)

Substituting, *dU = mCvdT* and *dW = pdV* in the above equation:

*dQ* = *mCvdT* + *pdV* + *dQ_loss_* (4)

Assuming the air–fuel charge inside the cylinder as an ideal gas, Equation (4) could be rewritten as given in Equations (8) and (9). 

The ideal gas equation is:

*pV* = *mRT* (5)

*pdV* + *Vdp* = *mRdT* (6)

*dT* = (*pdV* + *Vdp*)/*mR* (7)

(8) $$dQ=mC_{v}\frac{pdV+Vdp}{mR}+pdV+dQ_{loss}$$

(9) $$dQ=pdV\left(1+\frac{C_{v}}{R}\right)+\frac{C_{v}}{R}Vdp+dQ_{loss}$$

Since *C_v_/R = 1/(γ-1),* the Equation (9) is modified as given in Equation (10).

(10) $$dQ=pdV\left(\frac{\gamma }{\gamma -1}\right)+\frac{1}{\gamma -1}Vdp+dQ_{loss}$$

With consideration of convective heat transfer as major heat loss from the engine system, the Equation (10) could be written as given in Equation (11).

(11) $$dQ=pdV\left(\frac{\gamma }{\gamma -1}\right)+\frac{1}{\gamma -1}Vdp+h_{c}\;dA_{cylinder}\;d\left(T_{g}-T_{w}\right)$$

The Equation (11) could be expressed as Equation (12) for engine operation with respect to degree CA (HRR with respect to degree CA).

(12) $$\frac{dQ}{d\theta }=\frac{\gamma }{\gamma -1}p\frac{dV}{d\theta }+\frac{1}{\gamma -1}V\frac{dp}{d\theta }+h_{c}\frac{dA_{cyl}}{d\theta }\frac{d\left(T_{g}-T_{w}\right)}{d\theta }$$

## 3. Results and Discussions

### 3.1. Comparison of Performance Characteristics of the Engine with Different Fuel Blends

**Figure 2:**
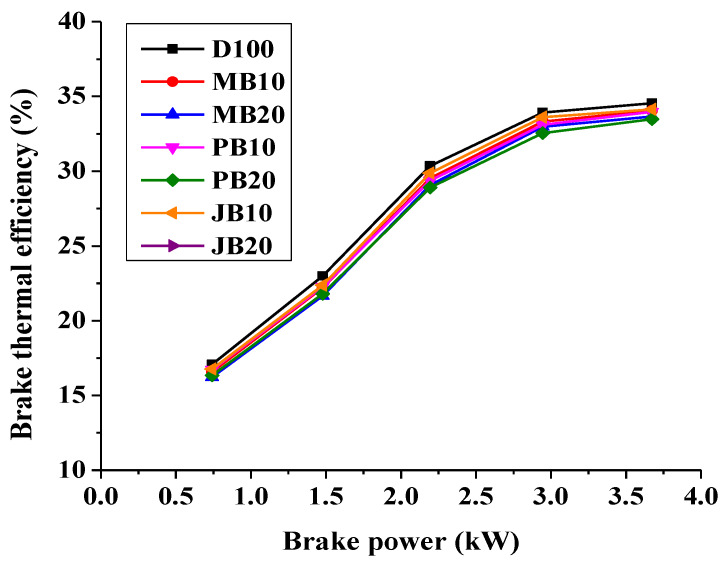
Comparison of brake thermal efficiency with respect to engine loading for microalgae, polanga, and jatropha biodiesel blends.

Figure 2 shows variation in brake thermal efficiency (BTE) of microalgae, jatropha, and polanga biodiesel blended fuels (MB10, MB20, JB10, JB20, PB10 and PB 20) with variation of engine loads compared to diesel (D100). The BTE of the engine with microalgae biodiesel blends (MB10 and MB20) was found better than other tested biodiesel blends (JB10, JB20, PB10 and PB20). The BTE increased significantly with increasing engine load with all types of fuel blends. However, at a particular load, the efficiency decreased slightly with biodiesel blending. For example, at 100% load (rated load), the BTE decreased from 34.55% with diesel to 34.05%, 33.66%, 33.91%, 33.47%, 34.13%, and 34.2% with MB10, MB20, JB10, JB20, PB10, and PB20 respectively. It may be noted that, under uncertainty limits, the efficiency of the engine with all biodiesel blend fuels was almost comparable with neat diesel operation. This may be due to lower calorific value of the biodiesel blended fuels than the base fuel (pure diesel). Calorific value of diesel, MB10, MB20, JB10, JB20, PB10, and PB20 fuels were examined in laboratory as 44.24 MJ/kg, 43.81 MJ/kg, 43.12 MJ/kg, 43.71 MJ/kg, 43.01 MJ/kg, 43.6 MJ/kg, and 42.94 MJ/kg. Similarly, it is to be noted that density of biodiesel blends (MB10: 833.5 kg/m^3^, MB20: 835.2 kg/m^3^, JB10: 832.8 kg/m^3^, JB20: 835.1 kg/m^3^, PB10: 832.9 kg/m^3^ and PB20: 834.8 kg/m^3^) are higher than diesel (830.1 kg/m^3^). Higher density, viscosity, and lower calorific value of fuel affects its spray characteristics such as poor atomization, high amount of heat absorption for vaporization of large fuel droplets and poor mixing of the fuel vapor, which lead to incomplete combustion reaction and results in higher fuel consumption [23,33,34]. 

**Figure 3:**
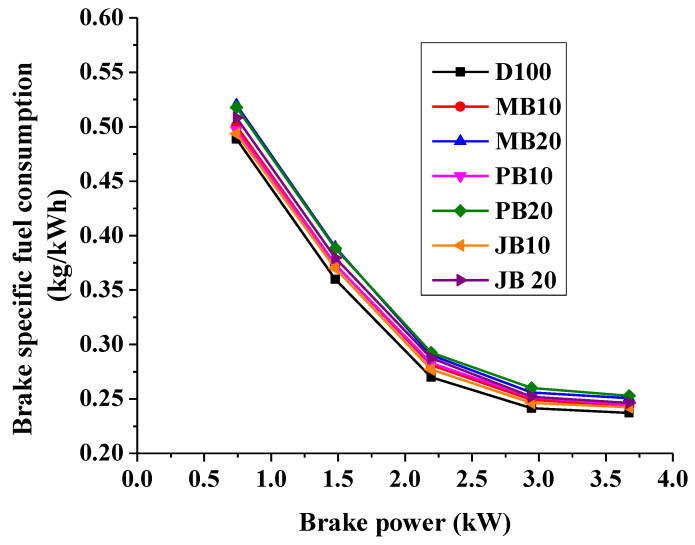
Comparison of brake specific fuel consumption with respect to engine loading for microalgae, polanga, and jatropha biodiesel blends.

Islam et al. also reported that the brake specific fuel consumption (BSFC) of algae biodiesel blend (MB50: 50% algae biodiesel and 50% diesel) increased by 9.3% in comparison to pure diesel [23] and therefore, the BTE of the CI engine decreased significantly with all fuel blends [23,25,35]. Similarly in another study, the BTE of a CI engine fueled with pure diesel fuel and microalgae biodiesel blends decreased from 30% (pure diesel) to 25% (MB20) [25]. The variation in brake specific fuel consumption (BSFC) of the test engine fueled with all biodiesel blends (MB10, MB20, JB10, JB20, PB10, and PB20) are shown in Figure 3. As shown in Figure 3, the BSFC of the test engine was found to be decreased with increasing load. In addition, the fuel consumption of the tested engine was also increased with increasing biodiesel blending share from 10% to 20%. For example, at 100% load, BSFC increased from 0.237 kg/kWh with pure diesel to 0.243 kg/kWh, 0.250 kg/kWh, 0.244 kg/kWh, 0.252 kg/kWh, 0.242 kg/kWh, and 0.246 kg/kWh with MB10, MB20, JB10, JB20, PB10, and PB20 fuels respectively. It could also be observed that the BSFC increased with higher rates at lower load (20% load) than at higher load (100% load) as shown in Figure 3. However, BSFC for all fuel blends are almost comparable with neat diesel operation under uncertainty limits. Wahlen et al. also observed that the BSFC of a multicylinder 7.9 kW CI engine also decreased with a microalgae-biodiesel blend [26]. In a comparative assessment of microalgal oil with croton oil, Tsaousis et al. reported that microalgal biodiesel demonstrated significantly higher BSFC at lower loads (10%) than higher loads [17]. In contrast, the higher oxygen content of microalgal biodiesel as compared to croton biodiesel leads to complete combustion of the fuel and thus higher in-cylinder temperature rise which finally lowers the BSFC at higher loads [17].

### 3.2. Comparison of Combustion Characteristics of the Engine with Different Fuels

**Figure 4:**
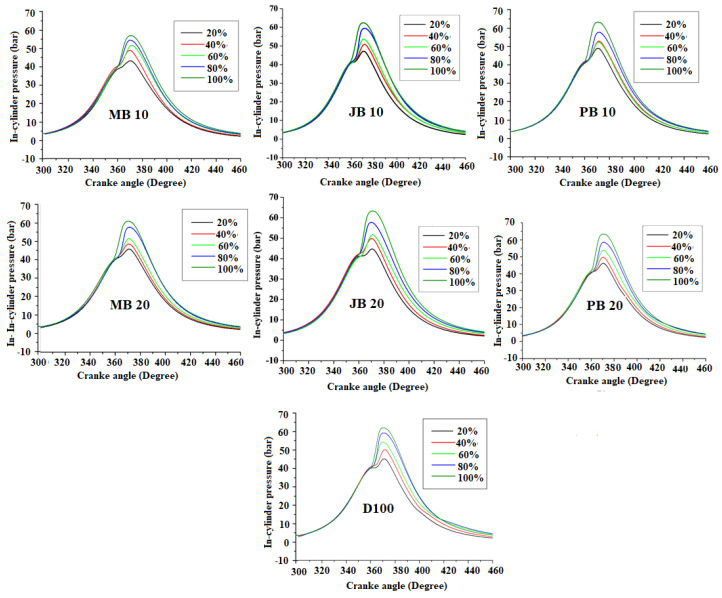
In-cylinder pressure profiles with diesel, microalgae, polanga, and jatropha biodiesel blends at different loads.

**Figure 5:**
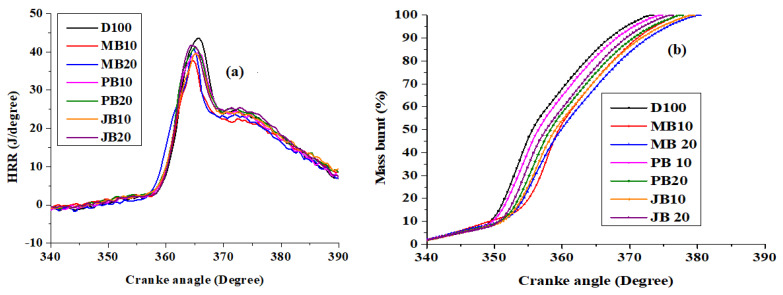
(**a**) Heat release rate profiles with diesel, microalgae, polanga, and jatropha biodiesel blends at 100% load. (**b**) Mass fractions burnt in the engine with diesel, microalgae, and polanga biodiesel blends at 100% load.

The in-cylinder pressure profiles and heat release rate (HRR) with respect to crank angle for all kinds of tested fuels (D100, MB10, MB20, JB10, JB20, PB10, and PB20) are shown in Figure 4; Figure 5 respectively. It was observed that the maximum in-cylinder pressures were about 63.3, 57.7, 60.9, 62.4, 63.1, 63.3, and 63.5 bar for D100, MB10, MB20, JB10, JB20, PB10, and PB20 fuel blends at 100% load. The in-cylinder pressure increased significantly with increasing engine load with all the biodiesel blends (MB10, MB20, JB10, JB20, PB10, and PB20). In addition, the peak pressures with jatropha and polanga biodiesel blends are higher than microalgae-based biodiesel blends Figure 4. The decreasing in-cylinder pressure trend could be due to reduction in combustion reaction rates. Experimental work carried out by Islam et al. also revealed the similar results of decreasing of in-cylinder pressure with algae biodiesel blends [23]. This could be attributed due to lower calorific value and higher viscosity [23]. Ignition delay could also be increased due to poor atomization of high-density fuel [36]. This effect is even worse with high percentage of biodiesel blending shares and subsequently, the in-cylinder pressure reduced at higher rates with MB20 as compared to MB10.

The heat release rate (HRR) of the engine with respect to degree crank angle at 100% load is illustrated in Figure 5a. The HRR increased significantly with increase in the engine load. However, for a particular load, the heat release rates with all biodiesel blends are lower than the pure diesel operation. For example, at the rated load, peak HRR decreased from 43 J/°CA with base diesel to 37, 40, 39, 41, 39, and 40 J/°CA with MB10, MB20, JB10, JB20, PB10, and PB20 fuels respectively (Figure 5a). It is evident from the results that the HRR is reduced with rising biodiesel share in the fuel blend considerably. With MB20 fuel, the cumulative heat release (CHRR) increased from 1095.9 J at 20% load to 1484.2 J, 1763.9 J, and 1837.8 J at 60%, 80%, and 100% loads respectively. However, the CHRR decreased significantly with MB10 and MB20 fuels as compared to pure diesel. It is well known that CHRR is an indicative measure of combustion efficiency of the engine [37,38]. Hence, reduction in the CHRR directly leads to decrease in combustion efficiency and subsequently reduction in thermal efficiency of the engine. Figure 5b represents mass fraction burnt with respect to degrees crank angle for all the biodiesel fuel blends at 100% load. The combustion process delayed considerably with the biodiesel blended fuels as shown in the Figure 5b. For example, 100% mass fraction burnt was achieved at 372.8 °CA with base diesel, but it was delayed to 380.1 °CA with MB20 fuel.

### 3.3. Comparison of Emission Characteristics of the Engine with Different Fuels

**Figure 6:**
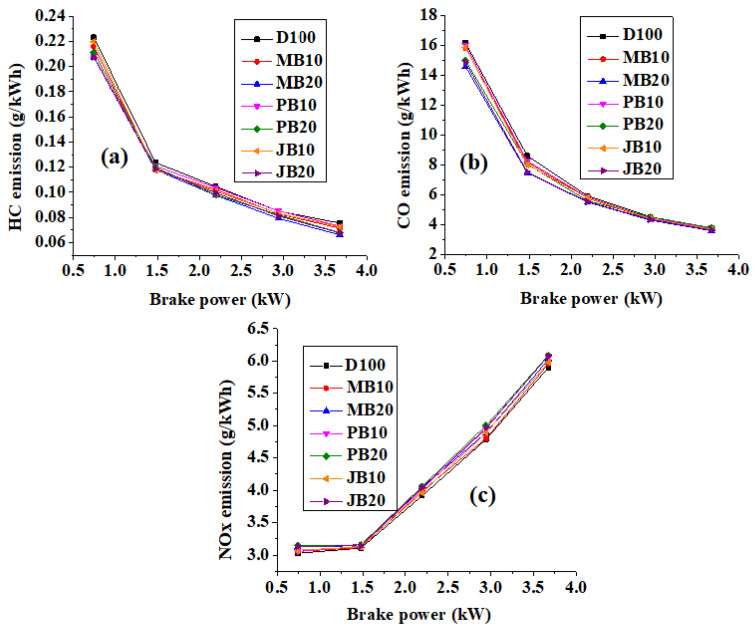
(**a**) Comparison of HC emissions with respect to engine loading for microalgae, jatropha, and polanga biodiesel fuels. (**b**) Comparison of CO emissions with respect to engine loading for microalgae, jatropha, and polanga biodiesel fuels. (**c**) Comparison of NOx emissions with respect to engine loading for microalgae, jatropha, and polanga biodiesel fuels.

Figure 6a,b illustrates a comparison of hydrocarbon (HC) and carbon monoxide (CO) emission characteristics of the tested engine powered with various biodiesel fuel blends. Both types of emissions decreased significantly for all biodiesel fuel blends with increasing biodiesel blending share at lower loads. However, at higher loads, the emissions reduction was marginal. For example, the HC emission decreased about 3.27%, 7.06%, 1.56%, 7.10%, 6.81%, and 5.29% for MB10, MB20, JB10, JB 20, PB10, and PB20 fuels, respectively, at 20% engine load, whereas it was 5.37%, 12.41%, 3.89%, 10.33%, 3.22%, and 9.99% lower for MB10, MB20, JB10, JB20, PB10, and PB20 fuels, respectively, at 100% load in comparison to base fuel (pure diesel). Similarly, the CO emissions were also reduced with increasing percentage of biodiesel in fuel blends. On average, the CO emissions for MB10, MB20, JB10, JB20, PB10, and PB20 fuels were decreased by 3.1%, 8.8%, 3.2%, 7.6%, 1.9% and 4.0% relative to neat diesel fuel. Additionally, it was also observed that CO emissions decreased with increasing load Figure 6b. This can be explained by the fact that biodiesel is an oxygenated fuel and the presence of oxygen in fuel blends leads to cleaner combustion which results in lower emissions [39]. The CO emission formation during combustion in CI engines is illustrated with the mechanism given in Equations (15)–(17) [39,40]. The CO emission decreased from 3.68 g/kWh, 3.61 g/kWh, 3.70 g/kWh, and 3.76 g/kWh with JB10, JB20, PB10, and PB20 fuel to 3.67 g/kWh and 3.57 with MB10 and MB20 fuels from the test engine at higher load. In addition, both the MB10 and MB20 fuels (third-generation biofuels) also showed lower CO emission levels than diesel (base fuel) and JB10, JB20, PB10, and PB20 (second generation fuels) at all loads (Figure 6b).

RH →R → RO_2_ → RCHO → RCO → CO (13)

CO + OH → CO_2_ + H (14)

where rate constant for this reaction is:

k_f_,_CO_ = 6.76 × 10^10^ exp(T/1102) cm^3^/gmol (15)

Figure 6c represents oxides of nitrogen (NO_x_) emissions for different biodiesel blends at different loads from the tested engine. The engine-out NO_x_ emission was found higher with all the biodiesel fuels (i.e., MB10, MB20, JB10, JB20, PB10, and PB20) as compared to base diesel fuel. For example, the NO_x_ emissions increased from 5.89 g/kWh with diesel fuel to 5.96 g/kWh (MB10), 6.02 g/kWh (MB20), 5.98 g/kWh (JB 10), 6.08 g/kWh JB (20), 6.07 g/kWh (PB10), and 6.13 g/kWh (PB20) at higher load (100% load). It is well known that the NO_x_ emission formation in a CI engine mainly depends on three parameters: (i) in-cylinder temperature, (ii) amount of oxygen present inside the combustion chamber, and (iii) combustion reaction time [41]. In addition to these parameters, it could also be proved that the emission formation rate will depend on the temperature rise during premixed combustion phase [42,43,44]. Large number of double bonds (more unsaturation) leads to higher flame temperature during premixed combustion, resulting in more NO_x_ formation [45]. Higher NO_x_ emission values in the present study could be due to the dominant effects of higher oxygen content and higher reaction time (higher combustion duration). The in-cylinder temperature reduction with biodiesel blending might have an insignificant effect on the emission formation. Tüccar et al. also found an increment in NO_x_ emission of a DI 89 kW diesel engine with algae biodiesel–diesel blend (20% microalgae biodiesel and 80% diesel) from about 700 ppm to 810 ppm due to presence of more oxygen content in the microalgae biodiesel [21]. Velappan et al. also revealed that the NO_x_ emissions increased from about 760 ppm (100% diesel fuel) to 855 ppm (20% microalgae biodiesel and 80% diesel) in a 5.2 kW CI engine at full load [24]. Jayaprabakar et al. also conducted the experimental tests on a CI and observed that the emissions increased 15.49% with algae biodiesel (710 ppm) as compare to diesel fuel (600 ppm) at 100% load and 23° BTDC fuel injection timing [25]. In another study at 100% load, NO_x_ emission increased by 22% when engine powered with 50% microalgae blends [23]. The NO_x_ formation of biodiesels depends on higher gas temperature at the end of combustion cetane numbers and oxygen content in biodiesel [45]. In addition to this, longer carbon chain length in microalgae biodiesel would also lead to higher adiabatic flame temperature and thus high NO_x_ emission formation [45]. Makareviciene et al. represented that the physical mechanisms of formation of incomplete combustion products (carbon monoxide: CO) inside the combustion chamber are opposite to the mechanisms of NO_x_ emission formation [22]. As a rule, engine-out CO emission decreases if NO_x_ emission increases, and vice versa [22]. Contrary to these results, some researchers reported lower NOx emissions with biodiesel blends than base diesel [26,28]. For example, Wahlen et al. found 24% lower NO_x_ with microalgae biodiesel blended fuel as compared to diesel fuel in a multi cylinder CI engine [26]. Mathimani et al. also reported the similar results of lower NO_x_ emission with microalgae biodiesel blends than diesel fuel [27]. At 100% load, the NO_x_ emission values were 356, 353 and 329 ppm for diesel, B10 and B20, respectively [46]. Finally, it could be concluded that, though very few research works reported a reduction in NO_x_ emission with biodiesel blending, many of the studies support the fact of increase in the emission due to dominant effect of inherent oxygen content in biodiesel fuel.

**Figure 7:**
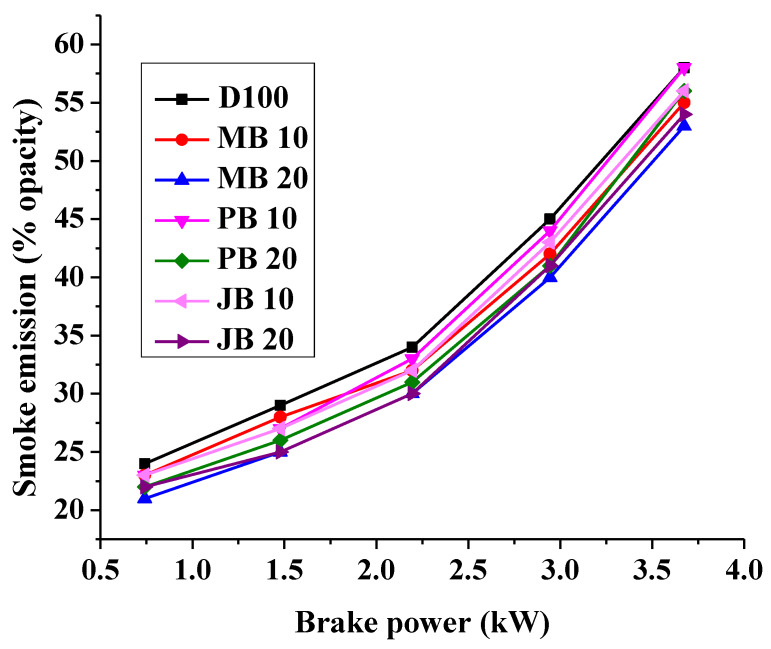
Comparison of smoke emission with respect to engine loading for different fuels.

The formation of smoke in CI engines takes place primarily due to heterogeneous combustion and the smoke opacity increases substantially with increase in degree of heterogeneity in the air–fuel mixture [41]. If the air–fuel ratio in a CI engine decreases, the smoke emission formation rate may increase drastically [38,43]. Smoke emission released from the tested diesel engine with different biodiesel blends at various load is represented in Figure 7. 

The results revealed that the smoke emissions increased significantly with increasing engine load for all fuel blends (D100, MB10, MB20, JB10, JB20, PB10, and PB20). For example, with MB20 fuel, the smoke emissions increased from 21% opacity at 20% load to 53% opacity at 100% load as shown in Figure 7. However, the smoke emission reduced considerably with increasing share of biodiesel in fuel blends. Many researchers reported that biodiesel has lower aromatics and extra oxygen in comparison to base fuel diesel [47,48]. The presence of low carbon molecules and oxygen leads to soot production in fuel and resulting into lower smoke emissions [34,49,50]. The lower sulphur content in biodiesel than base diesel fuels may be another reason behind this [33,47,49]. Furthermore, the smoke emissions also increased significantly with increases engine output power (load) for all tested fuel blends (diesel, MB10, MB20, JB10, JB20, PB10 and PB20). At rated load, the smoke emission reduced from 58% opacity with base diesel fuel to 55%, 53%, 58%, 56%, 56%, and 54% opacity with MB10, MB20, PB10, and PB20 fuels respectively (Figure 7). Smoke emission results in the current study are in line with the existing literature results. Makareviciene et al., in their experimental study, reported a reduction in smoke emission by 10% with algae biodiesel blend (30% biodiesel and 70% diesel) utilization in a 30 kW diesel engine [22]. Similarly, Dhamodaran et al. reported that smoke opacity was decreased with increasing biodiesel share in fuel blends [50]. High oxygen content, lower aromatics, and sulfur in the fuel blends reduces the possibility of soot precursor formation [45].

## 4. Conclusions

The experiments were performed on a single-cylinder 3.7 kW DI diesel engine with third-generation biofuels (MB10, MB20) and second-generation biofuels (JB10, JB20, PB10, and PB20) at various loads (20%, 40%, 60%, 80%, and 100%). The following conclusions emerged from the comparative assessment.

- The BTE of the engine decreased slightly with all the tested biodiesel blends (MB10, MB20, JB10, JB20, PB10, and PB20) in comparison to base diesel fuels at all loads, due to the lower calorific value, higher density, and viscosity of the biodiesel blends. However, this slight increase in the efficiency is insignificant under uncertainty limits.
- Lower in-cylinder pressure and heat-release rates were observed with the biodiesel blends due to slow oxidation reaction rates at low combustion temperatures. At 100% load, the in-cylinder peak pressure decreased from 63.3bar with base diesel to 57.7, 60.9, 62.41, 62.37, 63.3, and 63.5 bar for D100, MB10, MB20, JB10, JB20, PB10, and PB20 fuel blends at 100% load.
- All carbon-based emissions (hydrocarbons: HC, carbon monoxide: CO) decreased with increasing biodiesel blending share at all loads. This reduction was at higher rate at higher loads than lower loads. The HC emission decreased about 2.1%, 9.8%, 1.9%, 8.1%, 1.0%, and 7.5% for MB10, MB20, JB10, JB20, PB10, and PB20, respectively, at lower load. Similarly, engine-out CO emissions with MB10, MB20, JB10, JB20, PB10, and PB20 fuels decreased by 3.2%, 7.0%, 1.5%, 7.1%, 6.8%, and 5.2% relative to neat diesel fuel.
- NO_x_ emission increased with all the tested biodiesel blends as compared to base diesel fuel at all loads due to the dominant effect of oxygen content. However, NO_x_ emissions can be reduced significantly using engine after-treatment technologies such as selective catalytic reduction.
- Smoke emissions were found to be decreased marginally with increasing biodiesel blending share at all loads. At rated load, the smoke emission decreased from 58% opacity with base diesel fuel to 55%, 53%, 58%, 56%, 56%, and 54% opacity with MB10, MB20, JB10, JB20, PB10, and PB20 fuels respectively.

Finally, it could be concluded that microalgae-biodiesel blends are a more clean fuel for diesel engines than jatropha and polanga biodiesel, with a slight penalty in performance and emission characteristics of the engine. On the basis of the microalgae bio-oil yield per hectare per year, carbon dioxide sequestration and performance and emission characteristics, it can be concluded that microalgae (a third-generation fuel) is more suitable candidate to replace diesel fuel in CI engines in comparison to second-generation diesel fuels (jatropha and polanga biodiesel fuels).

## Figures and Tables

**Table 1:** Production of microalgae, jatropha, and polanga biodiesel.

S. No	Parameters	Jatropha Oil		Polanga Oil		Microalgae Oil	
		1st Stage	2nd Stage	1st Stage	2nd Stage	1st Stage	2nd Stage
1	Oil/lipid (mL)	250.0	276.5	250,0	268.4	250.0	265.1
2	Methanol (in %, *v/v* of oil)	50.0	25.0	50.0	25.0	50.0	25.0
3	Co-solvent (Toluene, % *v/v*)	1.0	-	1.0	-	1.0	-
5	Acid catalyst (Sulphuric acid, in %, *v/v*)	0.25	-	1.50	-	2.00	-
6	Base catalyst (Potassium hydroxide in%, wt/v)	-	0.9	-	1.1	-	1.5
7	Reaction temperature (°C)	64	65	64	65	64	65
8	Reaction duration (min)	7	6	10	8	12	15
9	Microwave power (watt)	700	700	700	700	700	700
10	Settling time (min)	45	70	45	70	45	70
11	**Results**						
	(a) Byproduct + impurities (glycerol + excess methanol + excess catalyst + gum), in mL	25.5 ± 2	-	32.6 ± 2	-	35.9 ± 2	68.3 ± 2
	(b) Methanol recovered	-	36 ± 3	-	33.7 ± 3	-	nd
	(c) Glycerol recovered (mL)	-	18 ± 2	-	15 ± 2	-	nd
13	Biodiesel Yield (in mL)	-	241.4	-	236.8	-	219.0
14	Final biodiesel Yield (%)		96.5 ± 3		94.7 ± 4		87.6 ± 3

Nd = not determined.

**Table 2:** Fuel properties of diesel and biodiesel blends.

Parameters	Test Method	Diesel	MB10	MB20	JB10	JB20	PB10	PB20	MB100	JB100	PB100	Error Analysis
**Density at 15 °C (kg/m^3^)**	ASTM-D 4052	830.1	833.5	835.2	832.8	835.1	834.1	836.9	889.0	878.0	883.0	±0.5%
**Viscosity at 40 °C (mm^2^/s) or cSt**	ASTM-D 445	2.85	2.91	2.99	2.92	3.01	2.94	3.03	4.87	4.98	5.24	±0.5%
**Pour point (°C)**	ASTM-D 97	−2	−3	−3	2	−1	4	−1	−4	1	3	±1%
**Flash point (°C)**	ASTM-D 93	57	88	101	90	108	89	120	154	162	158	±1%
**Copper strip corrosion**	ASTM-D 130	1	1	1	1	1	1	1	1	1	1	-
**Calorific value (MJ/kg)**	ASTM-D 130	43.94	43.34	42.74	43.71	43.01	43.23	42.52	38.94	36.94	38.08	±3%
**Oxidation stability (IP, at 140 °C, h)**	ASTM D 240-09	Not determined	16.3	7.3	18.3	8.6	14.8	6.9	4.5	3.7	3	±0.5%
**Water content**	ASTM D-2709	0.002	0.005	0.018	0.06	0.19	0.07	0.19	0.12	0.11	0.12	±0.5%

**Table 3:** Composition of microalgae, jatropha, and polanga biodiesel.

Fatty Acid Composition (%)	Carbon Structure	Composition		
		Microalgae Biodiesel	Jatropha Biodiesel	Polanga Biodiesel
**Capric acid**	C_10.0_	0.83	-	-
**Lauric acid**	C_12.0_	0.21	-	-
**Myristic acid**	C_14.0_	2.04	-	-
**Palmitic acid**	C_16:0_	21.26	15.87	11.97
**Palmitoleic acid**	C_16:1_	9.86	2.87	1.08
**Stearic acid**	C_18:0_	6.86	7.86	13.23
**Oleic acid**	C_18:1_	26.78	42.16	33.87
**Linoleic acid**	C_18:2_	31.08	32.14	36.34
**Linolenic acid**	C_18:3_	6.89	1.34	0.9
**Arachidic acid**	C_20.0_	0.29	-	-
**Erucic acid**	C_22.1_	2.18	-	-
**Saturated Fatty acids**		28.49	23.73	26.90
**Monounsaturated fatty acids**		35.44	42.03	34.95
**Polyunsaturated fatty acids**		35.97	33.48	37.24

**Table 4:** Technical specifications of the test engine.

Particulars	Specifications
**Engine type**	Single cylinder, four stroke, water cooled, direct injection compression engine
**Bore x Stroke**	80 × 110 mm
**Cubic capacity**	0.533 L
**Compression ratio**	16.5:1
**Rated speed (rpm)**	1500
**Power**	3.7 kW
**Specific fuel consumption**	245 g/kWh

**Table 5:** Detailed experimental test matrix.

Test Trails	Fuel Used	Engine Load	Measurements
Trail 1	Diesel	20% load: 0.7 kW 40% load: 1.5 kW 60% load: 2.2 kW 80% load: 2.9 kW 100% load: 3.7 kW	**Performance parameters:** Fuel consumption, Brake power **Combustion parameters:** In-cylinder pressure versus crank angle (CA) degrees **Emission parameters:** CO, HC, NO_x_ and Smoke emissions
Trail 2	MB10, MB20		
Trail 3	JB10, JB20		
Trail 4	PB10, PB20		

**Table 6:** Details of measurement range and resolution of instruments.

Instrument/Sensor Name	Measuring Parameter	Measuring Range	Resolution	Accuracy	Uncertainty (%)
NDIR analyzer	CO emission (by volume)	0–10%	0.01%	±0.03%	2.86
	HC emission (ppm)	0–10.000	1	±10	2.56
	NO_x_ emission (ppm)	0–5.000	1	<1000 ppm ± 5 ppm ≥1000 ppm ± 5% of value	2.692
Smoke meter	Smoke emission (%)	0–100% Opacity	0.1% Opacity	±1% of value	2.512
	BP				±2.2%
	BTE				±1.29%

## >Nomenclature

$\frac{\partial q}{\partial x_{1}},\frac{\partial q}{\partial x_{2}},...,\;\frac{\partial q}{\partial x_{n}}$	Partial differential of calculated parameter q which depends on different measured variables x_1_, x_2_,…,x_n._
$\dot{m}$	Mass flow rate of fluid/species/fuel, kg/s
$\dot{R}$	Universal gas constant, J/mol-K
MB	Microalgae-biodiesel
ASTM	American Standard Testing Method
BSFC	Brake specific fuel consumption, kg/kWh
BTE	Brake thermal efficiency, %
CHRR	Cumulative heat release rate, J/cycle
CI	Compression ignition
CO	Carbon monoxide
D	Diesel
F_species_	Mole fraction of exhaust gas species (CO, HC and NO_x_)
FFA	Free fatty acid
HC	Hydrocarbon emission, g/kWh
HRR	Heat release rate, J/⸰CA
JB	Jatropha-biodiesel
MW	Molecular weight
N	Engine speed, rpm
NDIR	Nondispersive infrared
NO_x_	Oxides of nitrogen, g/kWh
p	In-cylinder pressure
P	Rated power, kW
PB	Polanga biodiesel
Q	Heat release rate, kJ
R	Characteristic gas constant
T	Temperature, K or ^o^C
U	Internal energy, J
θ	Degree crank angle
